# The impact of cellular senescence and DNA damage on colorectal tumour progression

**DOI:** 10.1186/2051-1426-1-S1-P144

**Published:** 2013-11-07

**Authors:** Helen K Angell, Marie Tosolini, Bernhard Mlecnik, Gabriela Bindea, Tessa Fredriksen, Lucie Lafontaine, Maximilian Waldner, Franck Pagès, Viia Valge-Archer, Jérôme Galon

**Affiliations:** 1 Laboratory of Integrative Cancer Immunology, INSERM U872, Paris, France; 2 Université Paris Descartes, Paris, France; 3 Cordeliers Research Centre, Université Pierre et Marie Curie Paris 6, Paris, France; 4 University of Erlangen-Nuremberg, Erlangen, Germany; 5 Immunology, Georges Pompidou European Hospital, Paris, France; 6 MedImmune Ltd, Cambridge, UK

## 

We have previously defined the immune contexture as the type, density, location and functional orientation of tumour infiltrating immune cells, associated with patient prognosis in colorectal cancer (CRC). Here we describe potential mechanisms, including the DNA damage response (DDR) and cellular senescence, as contributing factors in shaping this intra-tumoural immune reaction. Cellular senescence, permanent proliferation arrest, is triggered by exogenous or endogenous stress, including telomere shortening, DNA-damage or oncogene activation. We have evaluated the expression of 53BP1, pATM and p16INK4a in a cohort of 205 CRC patients. In addition, telomere length and senescence-associated β-galactosidase have been analysed. A large panel of immune markers, including CD3, CD45RO, CD8 and CD57, were evaluated immunohistochemically. Patients who had increased DDR and senescence correlated with improved prognosis, both disease free and overall survival. Patients with high 53BP1 and pATM had significantly higher numbers of infiltrating immune cells at both the tumour centre and invasive margin. Functional experiments, including a transwell chemotaxis system, have illustrated that immune cells migrate towards soluble factors produced by senescent tumour cells. Our data provide further mechanisms resulting in changes of specific immune cell densities within the tumour, illustrating links between senescence and aspects of the DDR with the control of the anti-tumour immune response, and thus CRC disease progression.

